# Multiple novel filamentous phages detected in the cloacal swab samples of birds using viral metagenomics approach

**DOI:** 10.1186/s12985-021-01710-0

**Published:** 2021-12-06

**Authors:** Jian Zeng, Yan Wang, Ju Zhang, Shixing Yang, Wen Zhang

**Affiliations:** School of Medicine, Jiangsu University, 301 Xuefu Road, Zhenjiang, 212013 Jiangsu People’s Republic of China

**Keywords:** Viral metagenomics, Cloacal swab, Filamentous phage, *Inoviridae*, Virus evolution

## Abstract

Members of the family *Inoviridae* (inoviruses) are characterized by their unique filamentous morphology and infection cycle. The viral genome of inovirus is able to integrate into the host genome and continuously releases virions without lysing the host, establishing chronic infection. A large number of inoviruses have been obtained from microbial genomes and metagenomes recently, but putative novel inoviruses remaining to be identified. Here, using viral metagenomics, we identified four novel inoviruses from cloacal swab samples of wild and breeding birds. The circular genome of those four inoviruses are 6732 to 7709 nt in length with 51.4% to 56.5% GC content and encodes 9 to 13 open reading frames, respectively. The zonula occludens toxin gene implicated in the virulence of pathogenic host bacteria were identified in all four inoviruses and shared the highest amino acid sequences identity (< 37.3%) to other reference strains belonging to different genera of the family *Inoviridae* and among themselves. Phylogenetic analysis indicated that all the four inoviruses were genetically far away from other strains belonging to the family *Inoviridae* and formed an independent clade. According to the genetic distance-based criteria, all the four inoviruses identified in the present study respectively belong to four novel putative genera in the family *Inoviridae*.

## Main text

The *Inoviridae* is a large family of non-enveloped, flexible filamentous viruses with circular, + ssDNA genomes of about 5.5 to 10.6 kb in size which encodes 7–15 proteins. The family *Inoviridae* is divided into 21 genera including *Affertcholeramvirus*, *Capistrivirus*, *Coriovirus*, *Fibrovirus*, *Habenivirus*, *Infulavirus*, *Inovirus*, *Lineavirus*, *Parhipatevirus*, *Primolicivirus*, *Psecadovirus*, *Restivirus*, *Saetivirus*, *Scuticavirus*, *Staminivirus*, *Subteminivirus*, *Tertilicivirus*, *Versovirus*, *Vicialiavirus*, *Villovirus*, and *Xylivirus* (www.ictv.global/report/inoviridae). They infect gram-negative bacteria by using a unique strategy of virion morphogenesis [1–4]. They adsorb to host bacterial pill that are thought to spontaneously resulting in the entry of the phage genome into the host cytoplasm. Following entry, filamentous phage genomes replicate through a rolling-circle mechanism. Progeny virions release from cells by extrusion without causing host lysis. Filamentous phage DNA can persist extra-chromosomally or integrate into the bacterial genome. The interaction between filamentous phages and their hosts is generally considered to be a symbiotic relationship, phage replication only causes a slight burden on the host, and in turn increases the virulence and toxicity of the host evolution. Most of our understanding of the biology of filamentous phages comes from *Escherichia coli* phages called Ff filamentous phages which were discovered in sewage samples in the early 1960s [5]. Ff filamentous phage is one of the early cloning vectors for DNA sequencing, and served as the workhorse of phage display technology for past 30 years [6, 7]. Other filamentous phages infected *Pseudomonas aeruginosa* were called the “Pf1-like” phages (Pf1, Pf4, Pf5, Pf6 and Pf7) and widespread among *P. aeruginosa* strains [8]. Pf4 has a key role in the overall structure, organized remodeling and seeding of mature biofilm. Filamentous phages existing in biofilm matrix are self-organized into viscous liquid crystal arrangement, which provides bacteria with enhanced surface adhesion and resistance to drying and antibiotics [9]. In addition, Pf phage may contribute to clinical outcomes in *P. aeruginosa* infection in patients with cystic fibrosis [10]. A large number of inovirus-like sequences were obtained from microbial genomes and metagenomes recently, but still have many filamentous phages need to be identified [11, 12]. Wild birds as one of the most abundant species carry a large number of viruses. Here, four novel filamentous phages divergent with existing members of the family *Inoviridae* were identified from viral metagenomics database of wild bird.

During 2018 to 2019, 43 cloacal swabs of wild and breeding bird samples were collected by using disposable absorbent cotton swabs from Hunan and Zhejiang province of China including 16 for Black Swans (Hunan, wild), 15 for Sliver Pheasant (Hunan, wild), and 16 for Toucan (Zhejiang, breeding). All of the birds were adults and the exact ages were unknown.

Viral metagenomics approach was used to characterize viral sequences in the fecal samples. Briefly, tips of swabs were immersed into 0.5 ml Dublecco's phosphate-buffered saline (DPBS) and vigorously vortexed for 10 min, then centrifuged at 15,000 × *g* for 10 min. Each supernatant was collected in a new 1.5 ml centrifuge tubes and stored at -80℃ until use. About 50 μl supernatant from each sample was pipetted and pooled into different sample pools. Three sample pools were generated based on the bird species. Sample pools were centrifuged at 12,000 × *g* for 20 min at 4℃ and then filtered through a 0.45-μm filter to remove eukaryotic and bacterial cell-sized particles. Filtrates were digested by DNase and RNase at 37℃ for 60 min. Total nucleic acids were then extracted using QIAamp MinElute Virus Spin Kit (Qiagen) according to the manufacturer's protocol. Three libraries were constructed using Nextera XT DNA Sample Preparation Kit (Illumina) and sequences using the Miseq Illumina platform with 250 bases paired ends with a distinct molecular tag for each pool. For bioinformatics analysis, resulting raw reads were debarcoded using vendor software from Illumina. An in-house analysis pipeline running on a 32-nodes Linux cluster was used to process the data. Reads were considered duplicates if bases 5 to 55 were identical and only one random copy was kept. Clonal reads were removed and low sequencing quality tails were trimmed using Phred quality score ten as the threshold. Adaptors were trimmed using the default parameters of VecScreen which is NCBI BLASTn with specialized parameters designed for adaptor removal. Bacterial reads were subtracted by mapping to the bacterial nucleotide sequences from the BLAST NT database using Bowtie2 v2.2.4. The cleaned reads were de-novo assembled by SOAPdenovo2 version r240 using Kmer size 63 with default settings [13]. The assembled contigs, along with singlets were aligned to an in-house viral proteome database using BLASTx (v.2.2.7) with an E-value cutoff of < 10^–5^, where the virus BLASTx database was compiled using NCBI virus reference proteome (https://ftp.ncbi.nih.gov/refseq/release/viral/) to which was added viral protein sequences from NCBI nr fasta file (based on annotation taxonomy in Virus Kingdom).

For phylogenetic analysis, the zonula occludens toxin (Zot) and major coat protein sequences of reference strains belonging to different genera of *Inoviridae* were downloaded from the NCBI GenBank database. Related protein sequences were aligned alignment program implemented in the CLC Genomics Workbench 10.0, and the resulting alignment was further optimized using MUSCLE in MEGA-X [14] and MAFFT v7.3.1 employing the E-INS-I algorithm [15]. Sites containing more than 50% gaps were temporarily removed from alignments. Bayesian inference trees were then constructed using MrBayes v3.2.7 [16]. The Markov chain was run for a maximum of one million generations, in which every 50 generations were sampled and the first 25% of Markov chain Monte Carlo samples were discarded as burn-in.

**Fig. 1:**
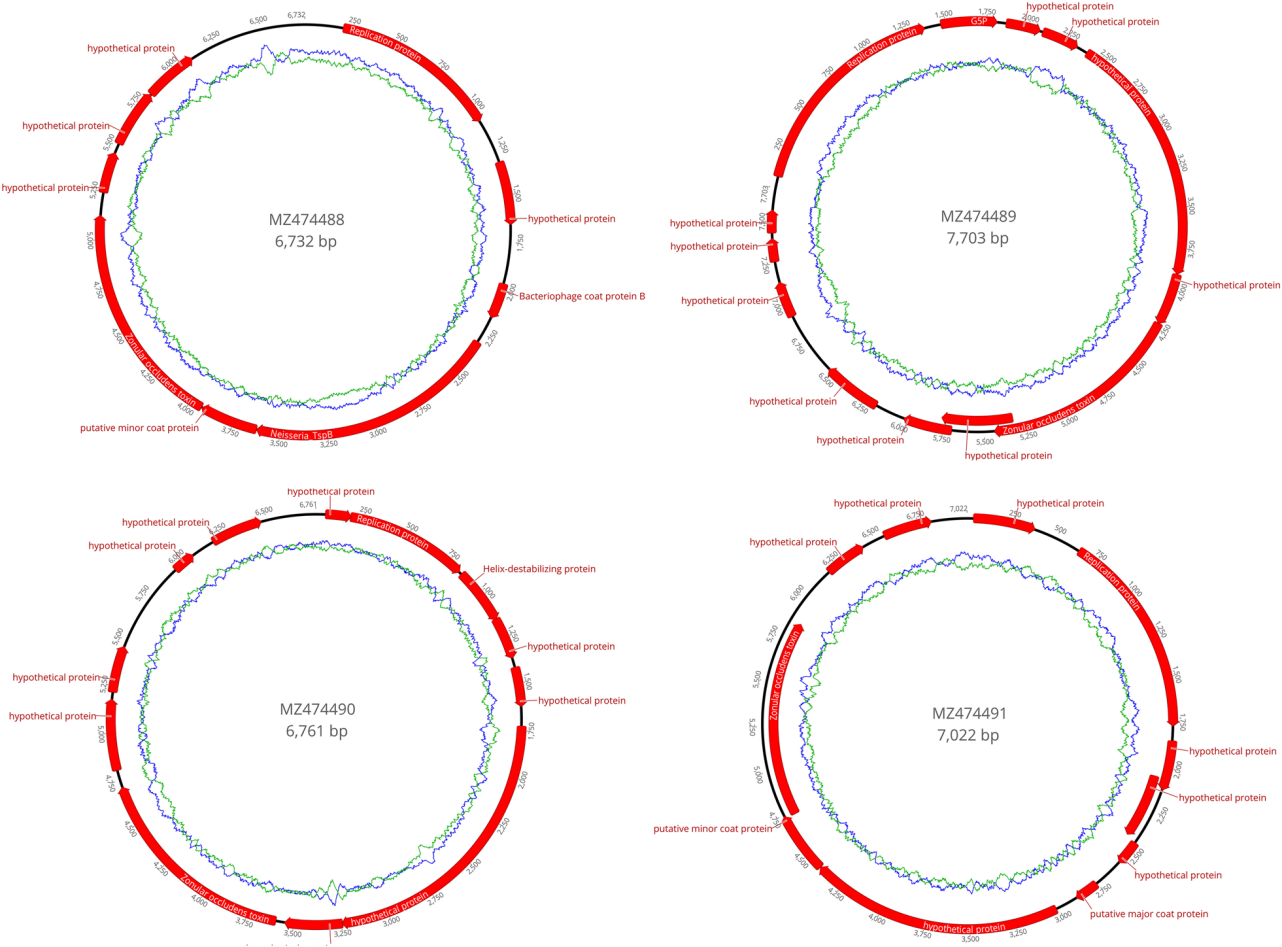
The genomic organization of four novel inoviruses was identified in this study. Viral encoding proteins were annotated, those proteins without homology to other proteins deposited in NCBI were named hypothetical proteins. The arrow indicated the direction of gene coding. The blue ring represents the GC content for selected sequences, while the green ring represents the AT content for selected sequence

Results showed that 599, 1236, and 1474 sequence reads respectively from 3 libraries belonged to the family *Inoviridae*. Four complete genomes of inoviruses were obtained by assembling using the low sensitivity/fastest parameter in Geneious 11.1.2 and named blackswan219-1, blackswan219-6, silverpheasant213, and toucan80 separately. Those four novel inoviruses were submitted to GenBank with accession no. MZ474488 to MZ474491. The genome of those four inoviruses are 6732 nt, 6761 nt, 7022 nt, and 7709 nt in length and have 9 to 13 open reading frames (ORF) respectively (Fig. 1). The GC contents of those four inoviruses are 56.5% for MZ474489, 54.3% for MZ474491, 51.4% for MZ474490, and 56.4% for MZ474488. Most encoding proteins of those inoviruses have no homology with other proteins deposited in NCBI database, while the replication protein (Rep) and Zot similar to other phages were detected in all those four inoviruses. The Rep of those four inoviruses shared amino acid sequence identity (98.3%, 76.5%, 94.4%, and 56.5%) to different Pseudomonas sp. deposited in NCBI. The Zot of those four inoviruses shared amino acid sequence identity (89.7%, 92.4%, 63.6%, and 30.3%) to different Pseudomonas sp. respectively. In addition, the putative major coat protein (CoaB) was only found in MZ474488 and MZ474491. Based on the above results, the Pseudomonas sp. may be the natural host of those four novel inoviruses and still need further study to testify it. Recently, analysis of metagenomic sequences has become an important means to study the diversity of viruses including eukaryotic viruses, bacterial viruses and archaeal viruses [17, 18]. Eugene V. Koonin et al., identified numerous genomes of virus-like elements that similar in size to tectiviruses and have diverse gene composition through searching tectivirus-like Double Jelly-Roll major capsid proteins in genomic and metagenomics sequence databases [19]. The Zot protein which conservative in the family *Inoviridae* can uses as the target for searching inoviruses in submitted metagenomics sequence databases.

**Fig. 2:**
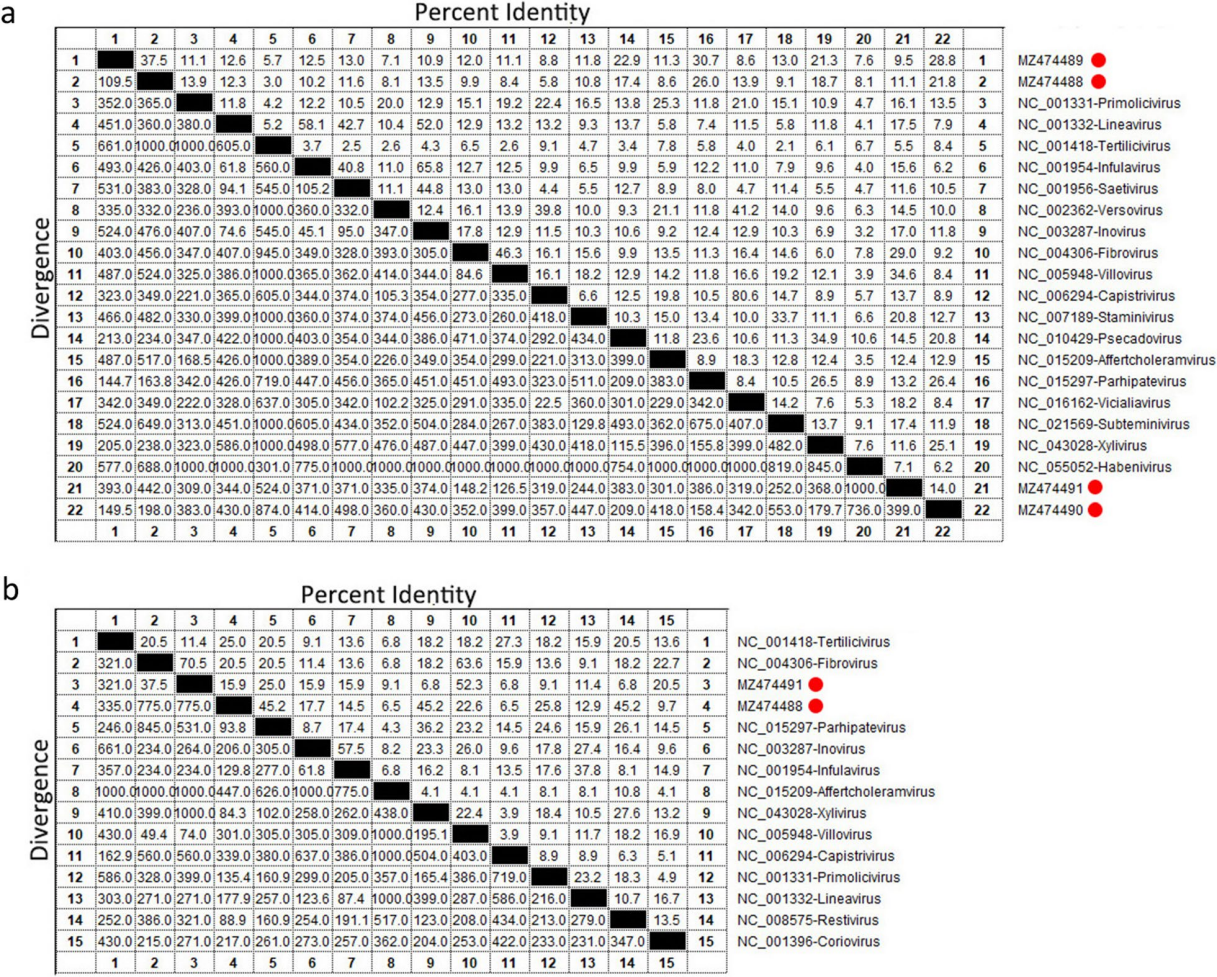
Sequence comparison of four novel inoviruses identified in this study. **a** Pairwise comparison of Zot amino acid sequences identified in this study with the representative strains of different genera of the family *Inoviridae*. **b** Pairwise comparison of CoaB amino acid sequences identified in this study with the representative strains of different genera of the family *Inoviridae*. Pairwise comparison was used Cluster W method which implemented into MegAlign program of DNAStar software. The “Percent Identity” and “Divergence” of sequences was calculated in default method and shown with picture

To determine the relationship between those four inoviruses and members of the family *Inoviridae*, the amino acid sequences of Zot and CoaB were compared and analyzed (Fig. 2). The result showed that the Zot proteins of all those four inoviruses shared the highest amino acid sequences identity (< 37.5%) to other reference strains belonging to different genera of the family *Inoviridae* and between each other (Fig. 2a), while the CoaB of MZ474488 and MZ474491 had the highest amino acid sequences identity of 45.2% and 70.5% to other reference strains (Fig. 2b). The ICTV states that inoviruses in different genera differ from each other by > 50% in the amino acid sequence of CoaB and Zot as assigned (ttps://talk.ictvonline.org/ictv-reports/ictv_online_report/ssdna-viruses/w/inoviridae). Based on the criteria, those four inoviruses identified here belongs to four novel genera of *Inoviridae*. In recent years, a genome organization-based taxonomy of prokaryotic viruses substituted for traditional morphology-based classifications to provide a relatively robust classification guide even for viruses with highly divergent genome sequences and organizations. Peter Simmonds et al., proposal that genomics-based classification of metagenomically derived viruses should be incorporated into the ICTV taxonomy in the future [20, 21].

**Fig. 3:**
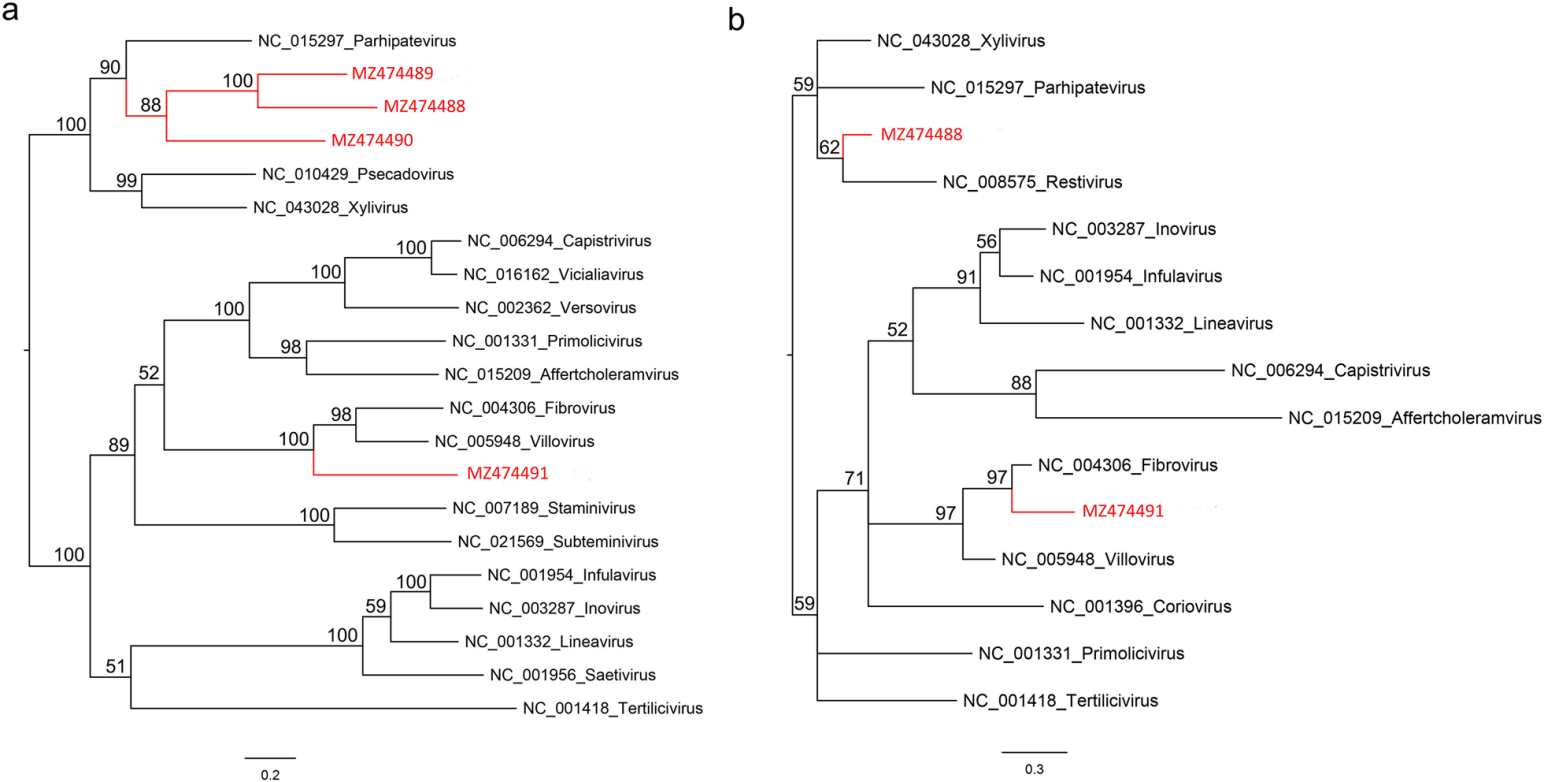
The phylogenetic analysis four novel inoviruses identified in this study. **a** The phylogenetic tree was constructed based on the amino acid sequences of Zot identified here, and reference strains of the family *Inoviridae*. **b** The phylogenetic tree was constructed based on the amino acid sequences of CoaB identified here, and reference strains of the family *Inoviridae*. Inoviruses identified in this study were marked with red. Scale bar indicates nucleotide substitutions per site. Branch support values for each branch are given

Two phylogenetic trees were constructed based on Zot and CoaB proteins including the reference strains of different genera of the family *Inoviridae* (Fig. 3). The result showed that all four inoviruses were forming an independent clade in the Zot tree separately (Fig. 3a). The strains MZ474489, MZ474488, and MZ474490 had a closer genetic distance with the strain NC_015297 of genus *Parhipatevirus*, while the strain MZ474491 had a closer genetic distance with the strain NC_005948 of genus *Villovirus*. Because the CoaB proteins of MZ474489 and MZ474490 failed to identify in this study, only the MZ474488 and MZ474491 showed in the CoaB tree. The strain MZ474488 had a closer genetic distance with the strain NC_008575 of genus *Restivirus*, but the strain MZ474491 had a closer genetic distance with the strain NC_004306 of genus *Fibrovirus* (Fig. 3b).

## Conclusions

In summary, we detected four novel filamentous viruses in fecal samples of wild and breeding birds and characterized their complete genome. All four inoviruses encoded the Zot proteins which were conserve in the members of the family *Inoviridae* and functioned as a key role in increasing the pathogenicity of lysogenic bacteria. Homology comparison based on the Zot proteins indicated that all those four inoviruses shared the highest amino acid sequence identity (< 37.5%) to other reference strains belonging to the family *Inoviridae* and among themselves. Phylogenetic analysis showed that all four inoviruses in this study were far away from other reference strains of different genera of the family *Inoviridae*. According to the genetic distance-based criteria, all four inoviruses belonged to four novel genera of the family *Inoviridae*. This study proved that viral metagenomics approach was suitable for the exploration and identification of filamentous phages.

## Data Availability

The genome of viruses obtained in this study were deposited in GenBank with the accession numbers: MZ474488 to MZ474491. The raw sequence reads from metagenomic library were deposited in the Shirt Read Archive of GenBank database under accession number: SRX7543785, SRX7544810, and SRX7545320.

## Abbreviations

Zot: Zonula occludens toxin
CoaB: Major coat protein
Rep: Replication protein
DPBS: Dulbecco's phosphate buffered saline
ORF: Open reading frame
BLAST: Basic local alignment search tool
